# Neurophysiological and Ultrasound Correlations in Guillain Barré Syndrome and CIDP—Case Series

**DOI:** 10.3390/jpm14060603

**Published:** 2024-06-05

**Authors:** Justyna Pigońska, Walkowiak Paweł, Marta Banach

**Affiliations:** 1 EMG Laboratory, Central University Hospital, Medical University of Lodz, 92-213 Lodz, Poland; juspigo@sck.um.pl; 2 Department of Neurology with Stroke Subdivision, John Paul II Regional Hospital of Belchatow, 97-400 Belchatow, Poland; p.walkowiak@szpital-belchatow.pl; 3 Department of Neurology, Jagiellonian University Medical College, 30-688 Krakow, Poland

**Keywords:** Guillain–Barré syndrome, chronic inflammatory demyelinating polyneuropathy, high resolution ultrasound, electroneurography

## Abstract

Introduction: Guillain–Barré syndrome (GBS) and chronic inflammatory demyelinating polyneuropathy (CIDP) are inflammatory polyneuropathies with an autoimmune etiology. These diseases differ mainly in the timing of their course but also in certain clinical differences. Electroneurography and electromyography are crucial for fulfilling the primary (for CIDP) and secondary (for GBS) diagnostic criteria. High-resolution ultrasound (HRUS) is recognized as a complementary method in the diagnosis of CIDP and GBS. Aim: The aim of this study was to present the neurophysiological and ultrasound findings of patients with clinically diagnosed inflammatory neuropathies (GBS and CIDP). Material and Methods: We collected data from clinically confirmed patients with GBS (3 persons) and CIDP (6 persons). The neurography and high-resolution ultrasound examinations according to the UPSS scale were performed. Results: The neurography tests of GBS and CIDP patients showed mainly demyelinating lesions of the examined nerves, often with abnormal F-wave recordings. Examination using HRUS in GBS patients showed mild and regional nerve swelling with hypoechoic bundles with a predilection for proximal segments and cervical spinal nerve roots. In contrast, CIDP patients had diffused nerve swelling with hypoechoic bundles of greater severity and extent than those with GBS. Conclusion: Neurophysiological tests and HRUS of peripheral nerves, plexi, and roots performed together can be very valuable, complementary diagnostic methods for the early diagnosis and effective treatment of inflammatory polyneuropathies.

## 1. Introduction

### 1.1. Guillain–Barré Syndrome (GBS)

Guillain–Barré syndrome is the most common cause of progressive flaccid quadriparesis. It occurs with a prevalence of 1–2/100,000 previously healthy individuals, with a predominantly male population [1,2]. It can be induced by infection prior to the disease [1,2] Zika virus and COVID-19 infection have been reported to correlate with GBS. Diagnostic criteria for GBS include symmetrical, progressive, usually monophasic, ascending limb paresis (sometimes initially only in the lower limbs) with abolition or weakness of tendon reflexes in the clinically involved limbs. Maximum symptom severity occurs up to week 4 of the disease, with a subsequent plateau and possible spontaneous (usually partial) improvement. Paresis may be accompanied by symmetrical sensory disturbances, initially, mainly distal [1,2,3]. There are several variants of GBS. In European countries, the classic, acute inflammatory demyelinating polyradiculoneuropathy (AIDP) form predominates, with a clinical course as outlined above. More severe variants include acute motor axonal neuropathy (AMAN; motor only) and acute motor and sensory axonal neuropathy (AMSAN), which are predominant in Asian countries and occur in approximately 20% of cases in Europe. Miller–Fisher syndrome (MFS) is characterized by a triad of symptoms (ophthalmoplegia, areflexia, and ataxia) and accounts for approximately 5% of all patients. Other forms, such as paraparetic, laryngopharyngeal, and Bickerstaff brainstem inflammation, are much less common [1,2,3]. In the classic form of GBS, the cranial nerves (especially the facial nerves) are often involved, and significant pain is experienced.

A serious complication of GBS is respiratory failure (25% of cases) [1]. The mortality rate in GBS is 3–7% and applies not only to the acute phase of the disease but also to the recovery period, especially in patients with autonomic system disturbances [1,2,3].

### 1.2. Confirmation of the Diagnosis Is Established on Clinical Symptoms

Cerebrospinal fluid examination with an increased protein level is very helpful in excluding other etiologies (e.g., infectious).

Electroneurography is an additional test used for assessing the type of nerve damage (axonal vs. demyelinating) and is indirectly helpful in determining prognosis. Characteristic changes include F-wave abnormalities, decreased conduction velocity, conduction blocks, prolonged terminal latencies in the case of axonal forms, and decreased response amplitude. These changes are predominant in motor fibers [1,2,3]. The presence of transient conduction disturbances, resembling the conduction blocks characteristic for AIDP, is also characteristic for AMAN.

In serological tests, diagnostic markers for the axonal forms include anti-GM1 and anti-GD1a antibodies. Anti-GQ1b antibodies are found in 90% of MFS cases [1,2,3].

### 1.3. Chronic Inflammatory Demyelinating Polyneuropathy (CIDP)

The symptoms of CIDP appear in patients of all ages, mainly between 40 and 60 years of age, more commonly in men. The incidence is 1/100,000, and the prevalence is 3–9 cases/100,000. A multi-year, slow, or relapsing course leading to increased restriction of mobility is characteristic [2,3,4].

Gait disturbance is a common complaint in the initial period. In CIDP, cranial nerve involvement and respiratory failure are rare. Multiple variants of CIDP have been described: Involvement of distal parts of the limbs (mainly lower limbs), multifocal with asymmetrical nerve involvement with predominance of the upper limbs, focal with involvement of one limb, and motor and sensory CIDP. The motor (paresis and muscle atrophy) and sensory (abnormal touch and vibration sensation with gait disturbance) forms are symmetrical and usually change over time to a mixed sensory-motor manifestation. Current diagnostic criteria highlight the greater prevalence of CIDP in diabetes mellitus, gammmapathy, and neoplastic and drug-induced diseases [2,3,4].

In CIDP, inflammatory changes in the myelin fibers and epineurium are accompanied by segmental demyelination of the peripheral nerves and possible subsequent remyelination and production of hypertrophic (bulbous) structures. These changes predominate in the proximal sections of nerves and progress with the duration of the disease. Macrophages are involved in the immunological process, and specific antibodies are directed against the adnexal region and nodes of Ranvier [5].

The criteria for CIDP are based on clinical features and neurophysiological abnormalities, including: (1) Progressive or recurrent proximal and distal paresis and sensory disturbances in at least 2 limbs (upper and lower), (2) duration of symptoms > 8 weeks, and (3) diminished or abolition of tendon reflexes in all limbs. The presence of antibodies directed against nodal and perinodal structures and the concentration of anti-MAG protein can support the diagnosis of CIDP.

High-resolution ultrasound and magnetic resonance imaging (MRI) are recommended as diagnostic procedures in CIDP. Nerve enlargement of at least two sites in the proximal median nerve segments and/or the brachial plexus supports the final diagnosis of CIDP. Ultrasound is a low-cost, widely available, non-invasive procedure. Its accuracy is considerably increased when using probes with a frequency >15 MHz. It allows for obtaining images of high resolution, which enables the accurate assessment of not only the main trunks of peripheral nerves but also the size and echogenicity of individual bundles. The distribution, size, and type of peripheral nerve lesions in polyneuropathies provide a recognized basis for directional differential diagnosis [6,7,8,9,10,11]. US utility is particularly high in inflammatory polyneuropathies, due to the fact that the nerve swelling observed by HRUS is primarily associated with demyelinating damage and is almost non-existent in cases of axonal lesions. In doubtful situations, T2-weighted MRI sequences with quantitative or semi-quantitative assessments of spinal nerve root sizes are also helpful [3]. The roles of evoked potentials and nerve biopsy are very limited in most patients; however, these can be performed in selected cases [6]. Using structured and again revised diagnostic criteria for GBS and CIDP and the continuously improving HRUS technique, the study analyzed cases of patients meeting clinical, neurophysiological, and ultrasound criteria [5,7].

## 2. Materials and Methods

Neurography and muscle examination, cerebrospinal fluid examination and a number of diagnostic tests to exclude other causes of the patients’ clinical condition were performed. Informed consent was collected from each patient. We collected data from 3 patients with GBS and 6 with CIDP. Electroneurographic tests and a standard neurological examination were performed. The electrophysiological examination was carried out according to standard procedures using a Dantec Keypoint Focus (Natus Medical Inc. Alpine Biomed ApS, DK-2740, Skovlunde, Denmark). Patients were subjected to an electrophysiological examination of the nerves in extremities most affected by the disease. For nerve stimulation, surface electrodes were used, and duration of the stimuli was 0.2 ms at all sites. Sensory nerve action potentials (SNAP) and compound motor action potentials (CMAP) were recorded with two surface plate electrodes situated 3 cm apart. For reference, previously established standards were used. Neurophysiological examination was performed according to the revision published European Academy of Neurology/Peripheral Nerve Society Guideline on diagnosis and treatment of Guillain–Barré syndrome from 2023 and the second revision of the 2021 European Academy of Neurology/Peripheral Nerve Society Guideline—electroneurography of the median and ulnar nerve (motor fibers, F-wave test, sensory fibers—orthodromic method), deep peroneal and tibial nerve (motor fibers and F-wave test), superficial peroneal and sural nerve (sensory fibers-orthodromic method) [5,7]. Electromyography was performed using a concentric needle electrode 37 mm × 0.46 mm. The scope of the examination was expanded to include other nerves until the features of demyelination or F-wave abnormalities consistent with accepted criteria were obtained. The selection of muscles that were subjected to EMG testing was also guided by the severity of the motor deficit. Differential diagnoses included congenital polyneuropathies, multiple mononeuropathies, motor neuropathy with conduction block and motor neuron diseases.

High-resolution ultrasound of peripheral nerves was performed using the 6–23 MHz linear probe of the Mindray Resona I9 diagnostic system (Mindray, Shenzhen, China). The Ultrasound Pattern Sum Score (UPSS) scale was used in the assessment of nerve swelling and cross-sectional area (CSA) measurements [8]. The examination protocol was identical for each patient. In the US evaluation of the brachial plexus, the long axis CSA of C5, and C6 roots and the CSA of the vagus nerve were included. In the upper limb, the median, ulnar, and radial nerves were evaluated. The course of the nerves was evaluated using the cable-car technique, with measurements made according to the points accepted in the UPSS [8]. For the median nerve, these were the wrist, elbow, and arm, while for the ulnar nerve, these included Guyon’s canal and arm. Lower limb nerves were assessed at the following points: tibial and peroneal nerves at the popliteal fossa, the tibial nerve at the tarsal canal, and the sural nerve at the calf [9,10].

The examination was carried out unilaterally, with the selection of the side according to the lateralization of symptom severity. Scoring of nerve swellings was performed according to the recommendations of the scoring system of the UPSS [3,4]. Nerve thickening between 100 and 150% of the defined CSA was rated with 1 point, while nerve thickening >150% of the defined CSA was rated with 2 points [9]. In addition, the type of peripheral nerve swelling under study was assessed using the Padua scale, which takes into account the distinction between class 1 swelling—thickened nerves and primarily hypoechoic bundles, class 2 swelling—thickened hypo and hyperechoic bundles, and class 3 swelling—no thickening and hyperechoic bundles [10].

## 3. Results

**Figure 1:**
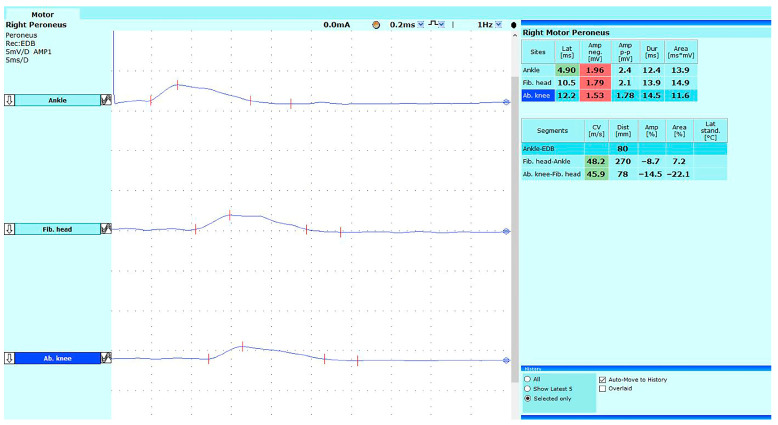
Electroneurography of the deep peroneal nerve with typical prolongation of duration of the motor action potential.

Neurography tests in GBS and CIDP patients mainly showed demyelinating damage to the examined nerves, often with a prolonged duration of CMAP (Figure 1) and abnormal F-wave recording. In this article, we have only included NCS results consistent with the standards for diagnosing demyelination according to the 2nd revision of EAN/PNS. On muscle examination, denervation was observed in acute cases, while reinnervation was seen in patients with CIDP.

Patients with confirmed GBS and a clinical suspicion for CIDP underwent HRUS examination. A characteristic distribution of nerve swelling was observed in both GBS and CIDP patients. For both groups of patients, the severity of swelling was greater in the extremities affected by a more severe motor deficit.

**Figure 2:**
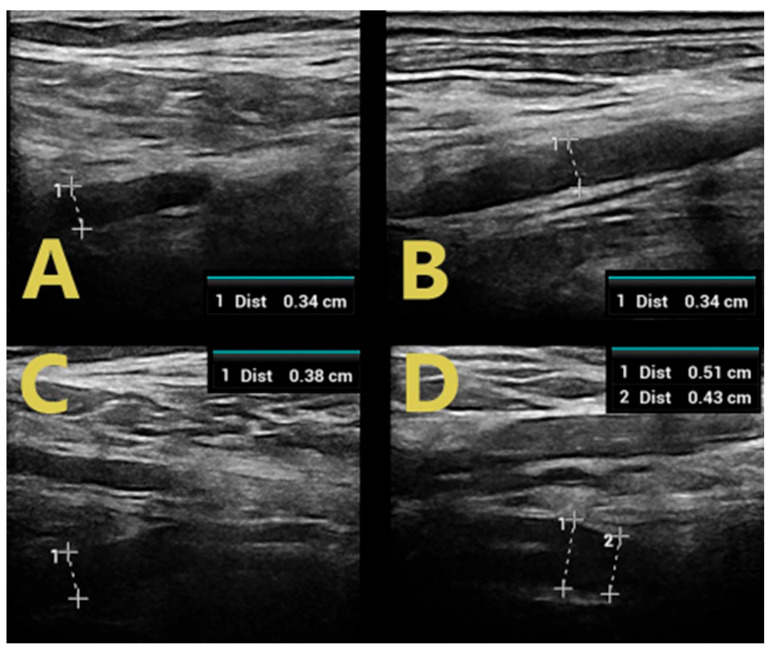
C5 roots in patients with GBS (**A**) and CIDP (**B**), C6 roots in patients with GBS (**C**), and CIDP (**D**).

**Figure 3:**
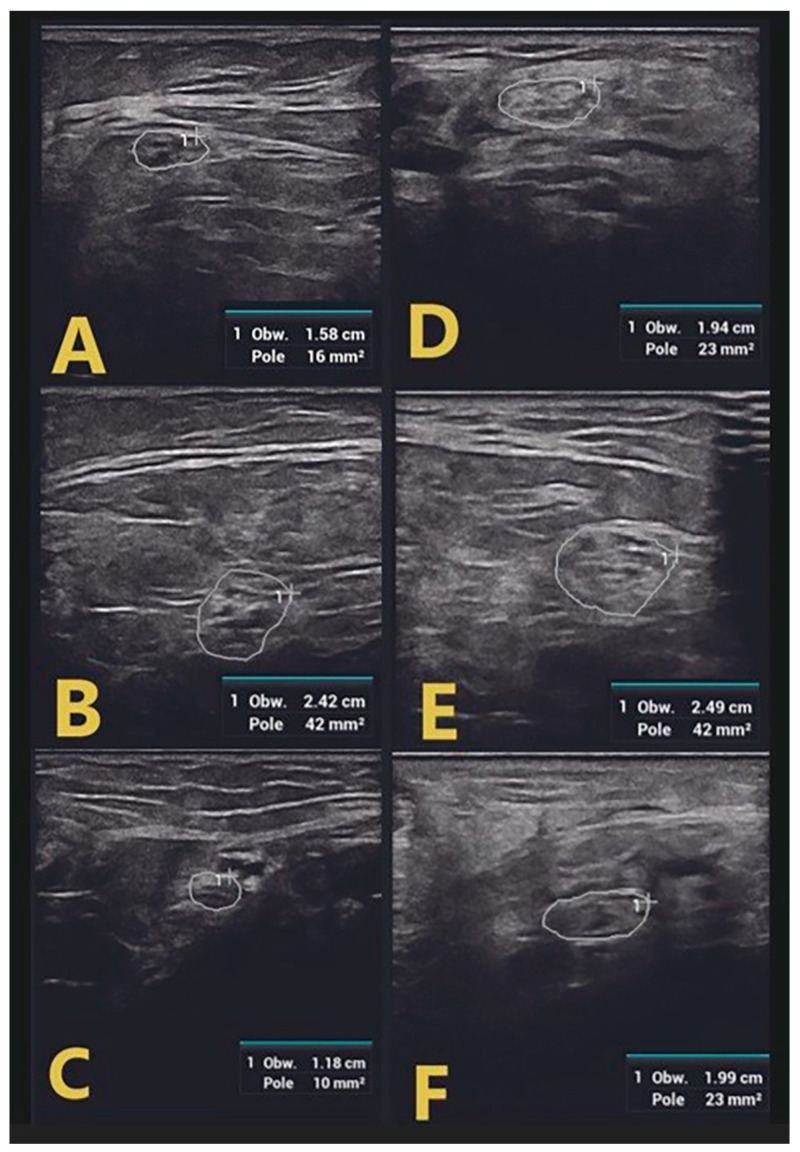
Swelling of the peroneal nerve in patients with GBS (**A**) and CIDP (**D**) and tibial nerve in the popliteal fossa ((**B**)—GBS, (**E**)—CIDP). A greater extent of tibial nerve swelling in the tarsal canal in a patient with CIDP (**F**) when compared to a patient with GBS (**C**).

Examination of GBS-confirmed patients revealed mild and regional nerve swelling with mostly hypoechoic bundles (class 1) with a predilection for proximal segments and cervical spinal nerve roots (Figure 2). In the lower limbs, swelling of the tibial and peroneal nerves was visualized only in proximal sections (in the popliteal fossa), with a normal image of the tibial nerve in the tarsal canal (Figure 3).

**Figure 4:**
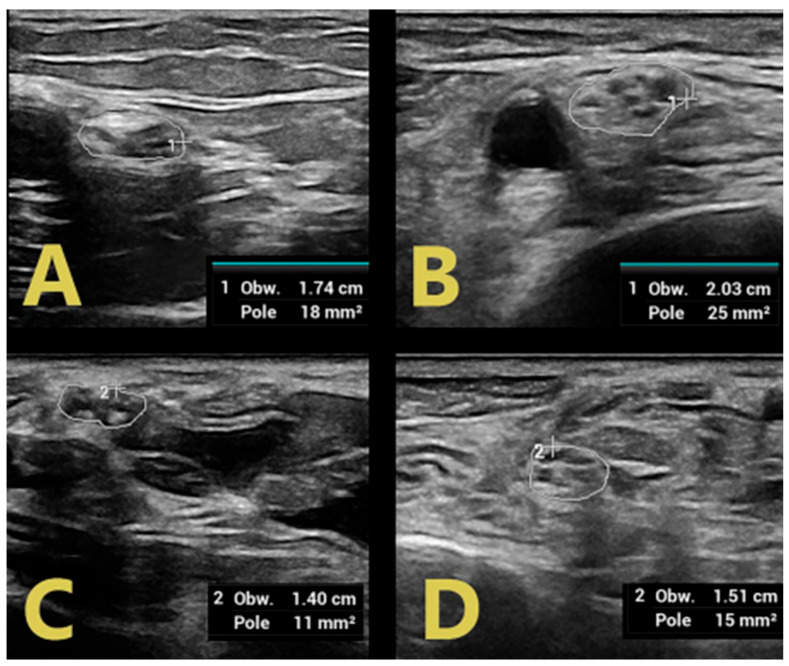
Swelling of the median nerve limited to the level of the arm in GBS (**A**) with no pathology at the level of the wrist (**C**) and a greater extent of nerve damage in CIDP ((**B**)—level of the arm, (**D**)—level of the wrist of the median nerve).

In the upper extremities, swelling involving the affected nerves was observed in multiple segments (Figure 4).

In contrast, patients with CIDP were found to have diffuse nerve swelling with hypoechoic bundles of greater severity and extent than those with GBS. Class 1 and class 2 nerve swelling was found in both the popliteal fossa and the tibial nerve in the tarsal canal in all patients with flaccid paraparesis of the lower extremities (Figure 2). In the upper extremities, swelling involving the affected nerves also involved multiple segments. Moreover, the severity of these swellings was greater than in GBS patients. This is probably related to the duration of the disease and chronic demyelination alternating with remyelination, leading to structural changes within the nerve bundles [11].

**Figure 5:**
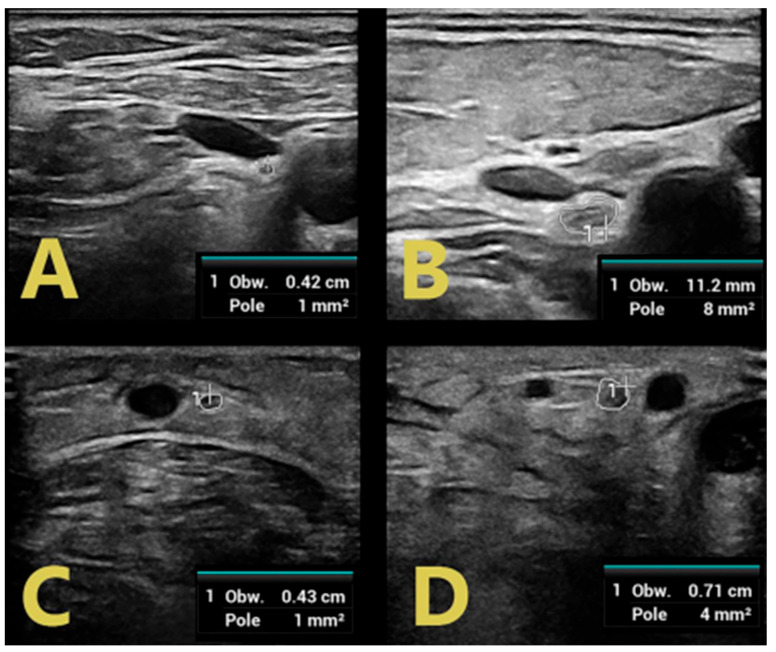
Normal image of the vagus nerve (**A**) and sural nerve (**C**) in GBS in comparison with swelling of vagus nerve (**B**) and sural nerve (**D**) in CIDP.

In addition, each CIDP patient showed thickening of the vagus and sural nerves; however, this was not seen in GBS patients (Figure 5). All data are presented in Table 1, Table 2 and Table 3.

## 4. Discussion

Chronic inflammatory demyelinating Polyneuropathy has a very heterogeneous presentation and consists of a spectrum of autoimmune diseases of the peripheral nerves. It is based on dysfunction resulting in autoimmunity against nerve antigens. The best examples are the recently discovered nodal and paranodal neuropathies that are caused by autoantibodies. Some autoantibodies are directed to axonal nodal structures, such as against Contactin 1 (CNTN1). Autoimmunity is primarily associated with axonal antigens, but not only with myelin antigens. Serological methods confirming the presence of antibodies targeting antigens of peripheral nerve structures, especially nodal and paranodal segments, are currently very valuable diagnostic tools. Approximately 25% of CIDP patients have circulating autoantibodies against antigens of peripheral nerve structures. Approximately 10% of patients have antibodies against neurofascin 186 contactin-1 and contactin-associated protein 1 (CASP1). Serological evaluation should be performed, especially in cases of acute and progressive CIDP initially classified as GBS and having a poor response to immunoglobulin [6]. Furthermore, the finding of antibodies against myelin-associated glycoprotein (anti-MAG) in patients with the distal form of CIDP may suggest future development of IgM paraproteinemia and monoclonal gammopathy. Because of this, attention should be paid to patients with acute onset who have coexisting ataxia, severe pain, and tremor [6].

The electrophysiological criteria for CIDP mainly emphasize the physiological effects of demyelination; however, in the context of possible paranodal and nodal pathomechanisms, a conduction block can also be observed.

In the context of CIDP, conduction block can result from paranodal abnormalities of the myelin sheath and also from primary dysfunction of the axon at the nodes of Ranvier. However, demyelination detected during EMG examination may be secondary to axonal damage (e.g., in the case of radicular lesions) or may be associated with damage in the course of mononeuropathy (e.g., in tunnel syndromes). The presence of a block should be differentiated from technical problems (e.g., with stimulation of the tibial nerve in the popliteal fossa or different nerves at Erb’s point) or the presence of anatomical variants such as Martin–Gruber anastomosis [6].

Imaging methods such as HRUS and MRI can be used to evaluate nerve abnormalities in their proximal segments and to visualize the enlargement of roots and the brachial or lumbosacral plexus. These imaging methods can also be used to avoid a misdiagnosis of demyelination or conduction block. Although MRI (revealing hyperintensity and enhancement after gadolinium administration) can be costly and time-consuming, it can help to localize and evaluate typical abnormalities at the site of the nerve lesion. Magnetic resonance imaging is particularly useful in the localization of root and plexus nerve swelling in places that are inaccessible to electroneurography and ultrasonography [6]. Using HRUS is very helpful; however, it is important to remember about misdiagnoses associated with a similar pattern of abnormalities in cases of polyneuropathies due to vasculitis, diabetes mellitus, neuralgic amyotrophy, and hereditary demyelinating neuropathies.

Previous studies discussing outcomes regarding specific imaging methods mostly comprised small numbers of cases. However, cut-off values for the enlargement of nerves both in HRUS and MRI should be defined [6]. A study by Goede et al. included six patients with suspected inflammatory neuropathy who did not meet the neurophysiological criteria for CIDP in the extensive NCS protocol and showed good response to immunoglobulins. In that study, peripheral nerve damage was confirmed by HRUS [13]. In a study by Herraets et al. comprising 38 CIDP patients, the sensitivity of ultrasound examinations was 97.4% and specificity was 69.4%, while the sensitivity of electroneurography was 76.9% and specificity was 93.5%. Additionally, the authors included eight patients in the cohort with a final diagnosis of CIDP. This group had normal NCS and abnormal HRUS and showed good response to IvIg or corticosteroid treatment [12]. Compared to the effectiveness of CIDP detection when using electroneurography alone, the additional use of HRUS improves diagnosis by 21.1% [14].

Work by Tan et al. showed that CSA is increased in CIDP and, to a lesser extent, in the demyelinating form of GBS—AIDP [15].

According to Grimm, who presented the neurophysiological and HRUS results of an analysis of 33 patients with GBS and 34 patients with acute CIDP, the positive predictive value for the diagnosis of GBS (with a sensitivity of 69.9% and a specificity of 85.3%) is the combination of the electrophysiological and ultrasound pattern of sural nerve sparing.

Nerve enlargement is the most important finding in acute and chronic immune-mediated neuropathies on ultrasound, in both acute and chronic types. Enlargement of the peripheral nerves, mainly sensory nerves and roots, predominates in CIDP compared to the transient enlargement of the roots and vagus nerve characteristic of GBS. Increased echogenicity is a specific (100%) feature of CIDP [16].

Recent guidelines published by the European Federation of Neurological Societies/Peripheral Nerve Society added prolonged duration of CMAP as a new criteria for the confirmation of CIDP [3]. In our group of CIDP patients, we observed abnormalities in four cases. Furthermore, the duration of CMAP was significantly prolonged in two GBS patients. Since this parameter can be the result of developing demyelination in polyradiculoneuropathy, it should be analyzed in every GBS patient. It can also indicate which nerve should be examined by HRUS more thoroughly. In our study, the GBS patient in the last position (LW) of the attached table (Table 2) underwent NCS examination. We observed an axonal-demyelinating lesion in the examined nerves; however, the results did not meet the full criteria for classical demyelination. The same nerves were subjected to HRUS examination. The size, type, and main distribution of the swelling and the UPSS score met the criteria for GBS, remaining consistent with the clinical manifestations, duration of the disease, and increased protein level in the cerebrospinal fluid. However, the massive swelling of the median nerve extended beyond the proximal segment typical for GBS. Because of this, monitoring US and NCS images over several months for the development of CIDP will be required. This may provide some data in considering whether HRUS can be used as an additional test to monitor the health and rehabilitation of patients with inflammatory polyneuropathies. Observing the obtained data, we collected more abnormalities in the HRUS examination, comparing the abnormalities and confirming neurophysiological criteria. Moreover, HRUS results were consistent regardless of the duration of symptoms, patients’ age, or neurophysiological findings and remained in close correlation with the clinical symptoms of the patients. The result of our observations is in line with reports regarding the effectiveness of HRUS in the diagnosis of inflammatory polyneuropathies, as it was stated earlier [8,9,10,11,13,14]. This is a suggestion for another discussion about increasing the sensitivity of neurophysiological criteria for CIDP and GBS [13]. Of particular note are patients with very early forms of GBS in whom the first early ENG examination rarely shows clear demyelinating changes [17,18]. The work of Herraets, Goedee, and Grimm addressed neurophysiological and ultrasound correlations based on previous CIDP criteria. Our work presents neurophysiological and ultrasound criteria based on the new, detailed 2021 neurophysiological guidelines [5,13,14,16].

Further advances in the diagnosis of CIDP will rely on the standardization of the evaluation of currently known autoantibodies and the demonstration of new autoantibodies and immune mechanisms. In the future, complete information, including neurophysiological, imaging, and serological data, may become more useful in standard clinical practice. However, abnormal results of performed tests should still be analyzed in the clinical context [14]. Precise diagnostic criteria allow for the inclusion of standard treatment for patients diagnosed with CIDP (IVIG vs. corticosteroids) and GBS (IVIG vs. plasma exchange) and for patients who do not meet the diagnostic criteria to consider other causes of neuropathy and include targeted treatment.

## 5. Conclusions

Neurophysiological tests and HRUS of peripheral nerves, plexus, and roots performed together can be very valuable complementary diagnostic methods for the early diagnosis of CIDP and GBS and for the initiation of effective treatment.

Cross-sectional areas are inversely correlated with motor conduction velocity. It is noteworthy that despite the convergence of the severity of motor deficits in patients with GBS and CIDP, when correlating lesions with the ENG examination, the degree and distribution of nerve swelling remained varied, which was an important differentiating factor between the two diseases [8,9].

## Figures and Tables

**Table 1:** Patients with diagnosed CIDP.

Patient Initials	Duration	Muscle Weakness MRC Scale		US Abnormality		ENG Abnormality	
		Upper Limbs	Lower Limbs	Upper Limbs	Lower Limbs	Upper Limbs	Lower Limbs
SM	>6 months	-	3/5 (ataxia)	↑ CSA RMN, RUN, SBRRN ↑ C5 LAX CSA ↑↑ C6	↑↑ CSA RTN, RPN, ↑ CSA RSN	↑ duration LMR	↑ duration RMP
				LAX CSA ↑ CSA VN			
DJ	17 months	-	3/5	↑↑ CSA LMN ↑ CSA LUN ↑ C5 LAX CSA ↑↑ C6 LAX CSA ↑ CSA VN	↑↑ CSA LTN, LPN, ↑ CSA LSN	↑ duration LMM	↑F latency LMT
DR	6 months	-	3/5	↑↑ CSA RMN ↑ CSA RUN ↑ C5 LAX CSA ↑↑ C6 LAX CSA ↑ CSA VN	↑ CSA RTN, RPN, RSN		↑ F latency RMT, LMT
SZ	>5 months	-	2/5	↑ CSA RMN, RUN ↑ C5 LAX CSA ↑↑ C6 LAX CSA ↑ CSA VN	↑↑ CSA RTN, RPN, RSN		F wave absence RMT, LMP
WJ	17 months	-	3/5	↑↑ CSA RMN ↑ C5 LAX CSA ↑↑ C6 LAX CSA ↑ CSA VN	↑↑ CSA RTN, RPN, RSN		↑ duration RMP, RMT
BA	6 months	4/5	3/5	↑↑ CSA RMN, RUN ↑ C5 LAX CSA ↑↑ C6 LAX CSA ↑ CSA VN	↑↑ CSA RTN, RPN, RSN	↑ duration RMU	↑ duration RMT

Clinical and MRS scale assessment, HRUS and electroneurography abnormalities. MRS—Modified Rankin Scale [12], HRUS assessment: RMN and LMN—right and left median nerve, RUN—right ulnar nerve, SBRRN—superficial branch of right radial nerve, LAX—long axis, RTN and LTN—right and left tibial nerve, RPN and LPN—right and left peroneal nerve, RSN and LSN—right and left sural nerve, VA—vagus nerve, ↑ CSA—increased cross-sectional area rated with 1 point, ↑↑ CSA—increased cross-sectional area rated with 2 points. Electroneurography abnormalities according to 2nd revision of EAN/PNS [5]: RMU—right motor ulnar nerve, LMP—left motor peroneal nerve, RMP—right motor peroneal nerve, LMM—left motor median nerve, LMT—left motor tibial nerve, RMT—right motor tibial nerve, RMR—right motor radial nerve.

**Table 2:** Patients with diagnosed GBS.

Patient Initials	Duration	Muscle Weakness MRC Scale		US Abnormality		ENG Abnormality	
		Upper Limbs	Lower Limbs	Upper Limbs	Lower Limbs	Upper Limbs	Lower Limbs
BD	14 days	-	2/5	↑ C5 LAX CSA ↑ C6 LAX CSA	↑ CSA LTN, LPN	↑ duration RMU, LMP	F-wave absence RMP
SMi	4 days	4/5	2/5	↑↑ CSA RMN ↑ C5 LAX CSA ↑ C6 LAX CSA	↑ CSA RTN,	↑ duration RMU	↑F latency RMP, LMP ↑ duration RMP, LMP, RMT
LW	6 weeks	-	3/5	↑↑ CSA RMN, ↑ RUN ↑ C5 LAX CSA ↑ C6 LAX CSA	↑ CSA RTN, RPN		

Clinical and MRS scale assessment, HRUS and electroneurography abnormalities. MRS—Modified Rankin Scale [12], Clinical and MRS scale assessment, HRUS and HRUS assessment: RMN and LMN—right and left median nerve, RUN—right ulnar nerve, SBRRN—superficial branch of right radial nerve, LAX—long axis, RTN and LTN—right and left tibial nerve, RPN and LPN—right and left peroneal nerve, RSN and LSN—right and left sural nerve, VA—vagus nerve, ↑ CSA—increased cross-sectional area rated with 1 point, ↑↑ CSA—increased cross-sectional area rated with 2 points. Electroneurography abnormalities according to 2nd revision of EAN/PNS [5]: RMU—right motor ulnar nerve, LMP—left motor peroneal nerve, RMP—right motor peroneal nerve, LMM—left motor median nerve, LMT—left motor tibial nerve, RMT—right motor tibial nerve, RMR—right motor radial nerve.

**Table 3:** Demographical and medical patients’ data.

	Age	Sex	Comorbidity
SM	45	M	DM 2, AH
DJ	59	M	DM 2, AH, DKD, bilateral THA, SC
DR	55	M	DM 2, AH, AF, MDD
SZ	66	F	DM 2, AH, FJA
WJ	85	M	DM 2, AH, AF, FJA, CHF,
BA	41	F	DM 2
BD	69	F	HA, CU, FJA, hyperthyroidism
SMi	41	M	No known comorbidity
LW	66	F	AF, surgical treatment of ASD

M—male, F—female, DM 2—type 2 diabetes mellitus, HA—arterial hypertension, DKD—diabetic kidney disease, THA—total hip arthroplasty, SC—skin cancer, MDD—major depressive disorder, FJA—facet joint arthropathy, CHF—chronic heart failure, CU—ulcerative colitis, ASD—atrial septal defect.

## Data Availability

The data presented in this study are available on request from the corresponding author. The data are not publicly available due to privacy.
